# Supplementary material for: Calcitriol Protects against Acetaminophen-Induced Hepatotoxicity in Mice
Source: Biomedicines. 2023 May 25;11(6):1534. doi: 10.3390/biomedicines11061534 (PMC10295301; doi:10.3390/biomedicines11061534)
Supplement: Supplementary file 1 [file biomedicines-11-01534-s001.zip › biomedicines-2385628-supplementary.pdf]

Supplementary Information

Supplementary Table S1. An organ index for the liver of mice in each group.

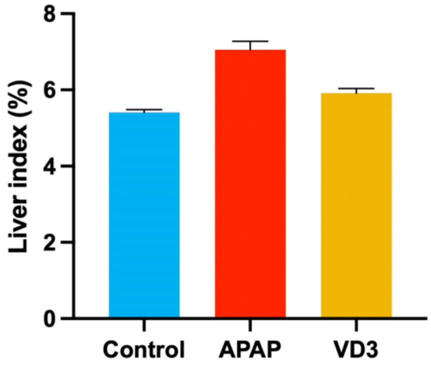

Group	Number	Mean body weight (g)	Mean liver weight (g)	Liver index (%)	p-value
Control	8	34.13 ± 0.64	1.85 ± 0.05	5.41 ± 0.07	<0.001
APAP	6	35.17 ± 2.64	2.48 ± 0.12	7.05 ± 0.22	
VD3	8	33.50 ± 1.69	1.98 ± 0.10	5.91 ± 0.12	
